# Factors affecting breast-feeding practice among a sample of Iranian women: a structural equation modeling approach

**DOI:** 10.1186/s13052-019-0724-9

**Published:** 2019-11-20

**Authors:** Reyhaneh Rafizadeh, Zahra Heidari, Mahmood Karimy, Fereshteh Zamani-Alavijeh, Marzieh Araban

**Affiliations:** 1 Student Research Committee of School of Health, Isfahan University of Medical Sciences, Isfahan, Iran; 2 Department of Biostatistics and Epidemiology, School of Health, Isfahan University of Medical Sciences, Isfahan, Iran; 3 Cardiac Rehabilitation Research Center, Cardiovascular Research Institute, Isfahan University of Medical Sciences, Isfahan, Iran; 4 Social Determinants of Health Research Center, Saveh University of Medical Sciences, Saveh, Iran; 5 Department of health education and promotion, School of Health, Isfahan University of Medical Sciences, Isfahan, Iran; 6 Department of Health Education and Promotion, Public Health School, Ahvaz Jundishapur University of Medical Sciences, Ahvaz, Iran

**Keywords:** Breastfeeding, Infants, Attitude, Social support, Self-efficacy

## Abstract

**Background:**

Breastfeeding is one of the most sensitive stages in the development of children, having many benefits for the mother and the child. The present study aimed to determine factors associated with breastfeeding intention and behavior in mothers living in Taft County, Iran.

**Methods:**

In this cross-sectional design, the statistical population consisted of 420 mothers with infants under 1 year of age living in Taft County of Yazd province, Iran. The research data were collected from health records of infants under one in health centers of the county as well as a researcher-made questionnaire including demographic information, knowledge and attitude towards breastfeeding, social support and perceived self-efficacy, and breastfeeding intention and behavior. Structural Equation Modeling (SEM) based on AMOS 18 were employed to analyze the relationship between research variables.

**Results:**

The mean age of mothers was 28.04 ± 6.49 year and the children were 10 ± 6 months. Half of the mothers (50.3%) had cesarean sections and more than half (55.8%) of the children were males. Mothers’ attitude (β = 0.442; 95% CI: 0.284, 0.599), self-efficacy (β = 0.186; 95% CI: 0.047, 0.324) and perceived social support (β = 0.178; 95% CI: 0.035, 0.322) were respectively the strongest predictors of breastfeeding intention. Besides, the breastfeeding intention affected breastfeeding behavior with a high coefficient (0.857; 95% CI: 0.735, 0.979).

**Conclusions:**

The study provided informative pathways on the association of maternal attitude, social support and self-efficacy with breast feeding behavior. These findings could be useful for designing health education and promotion programs about breast feeding among women.

## Background

Infancy is a sensitive stage in the children’s growth, and breastfeeding has many benefits for mothers as well [1]. Breast milk is an ideal and perfect food for infants, especially during the first few months of their life [2, 3]. Breast milk increases the infants growth, protects them against diseases, reduces mortality, and accelerates the process of disease treatment [4]. Besides, there is strong evidence regarding the association between breastfeeding and better growth and most cognitive, emotional and social aspects in children such as better intelligence and academic performance [5]. Previous studies have indicated that the lack of breastfeeding increases the risk of diseases such as respiratory infections, allergies, digestive problems, malnutrition, diabetes, obesity [6] and cancer in childhood and adolescence [3]. Improving child growth and reducing health costs through breastfeeding lead to economic gains for families and the country [7]. In addition, mothers who feed their children with breast milk are more likely to return to their pre-pregnancy body weight following delivery [8], and they run lower risks of diabetes, breast and ovarian cancer compared with non-breastfeeding mothers. Breastfeeding can help build a strong connection between mother and child, creating normal or positive feelings in mothers and children and preventing postpartum depression [9, 10].

The World Health Organization (WHO) recommends exclusive breastfeeding for 6 months [11]. Despite the global efforts to increase breastfeeding, this index has increased only by 6% in developing countries from 1995 to 2010 (from 33 to 39%) [12]. The improvement in breastfeeding may save the lives of five thousand children around the world every day [13]. Moreover, the WHO underscores its continuity until the age of 24 months, emphasizing the promotion of breastfeeding as one of the four strategies for child health [14]. Despite the benefits of breastfeeding, less than 40% of infants from 0 to 6 months of age have exclusive breastfeeding, and only about 15% are breastfed until 2 years of age [7]. In a study by Saffari et al., only 34% of mothers had exclusive breastfeeding up to 6 months in Iran [10].

According to the objectives of WHO, the identification of factors affecting breastfeeding is a strategy to increasing the amount of exclusive breastfeeding and manage programs for its promotion. Various modifiable factors can affect a mother’s intention for breastfeeding and its continuity, including the mothers’ skills and knowledge regarding breastfeeding, and their cultural, social and economic status, self-efficacy, and joy of breastfeeding, the supportive systems,, health personnel skills, breastfeeding immediately after delivery and psychological factors such as individual and familial beliefs [10, 15–17]. Studies indicate that mothers’ wrong knowledge and attitudes towards their abilities in breastfeeding, and the concern that they have inadequate milk for fulfilling their infants’ needs are the most important causes related to the non-continuity of breastfeeding [15, 18]. Results of a research by Kohan et al. indicated that the sense of breastfeeding adequacy in mothers was a key factor in its continuity depending on the ensured proper growth and health of child and the positive feedback from family and society; Moreover, informing mothers about the symptoms of insufficient breastfeeding and changing their wrong beliefs about breastfeeding led to an increase in breastfeeding [19]. In a meta-analysis by Nelson, mothers were concerned about their abilities to produce enough milk (low self-efficacy) to meet their infants’ needs [20].

Self-efficacy is affected by various factors such as social support, type of delivery, and mothers’ educational levels, understanding of the breastfeeding process, knowledge and attitude towards breastfeeding, and anxiety [15]. Social support is an important factor in breastfeeding, including support from spouse, friends and health care providers that play important roles in the onset and continuation of breastfeeding [21]. In this regard, the results of a study by Parsa et al. [22] indicated that maternal attitudes towards breastfeeding, social support and appropriate conditions for breastfeeding in society are important factors of successful breastfeeding following delivery. Self-efficacy is a psychological factor affecting the duration of breastfeeding.

Since exclusive breastfeeding is influenced by cultural factors, beliefs, norms, policies and attitudes of society [23], every society needs to pay attention to factors associated with breastfeeding and design a program to promote exclusive breastfeeding up to 6 months and its continuity, because a cultural factor may prevent exclusive breastfeeding in one region, but have no effects on another; thus, any region requires its educational orientation. In Iran, exclusive breastfeeding and its continuation is less than than recommended by WHO [15]. Efficacious breastfeeding depends on various socio-demographical and psychological variables. Accordingly, the present research aimed to study factors, such as self-efficacy, attitude, social support, and demographic variables of mother and infant with regards to the intention and behavior of breastfeeding in mothers with children under 1 year of age.

## Methods

### Participants and data collection

This is a cross-sectional study on 420 mothers with infants under 1 year of age in Taft County of Yazd province, Iran, which were selected through simple random sampling method (2018). A sample size of 345 participants was considered along with 34% breastfeeding prevalence [10], 5% precision with a two-sided 5% significance level, and 95% power. In addition, the dropout rate was estimated around 20%. Thus, the total sample in this research was 420.

The inclusion criteria included mothers who had children under one admitted to the health community centers of Taft County. The exclusion criteria were breast diseases, which prohibited feeding, a disease that interfered with breastfeeding, mothers’ use of antidepressants and psychedelic drugs, and the lack of informed consent to participate in the study. Mothers were selected follows: first, a list of all mothers with children under one was obtained from Taft Health Centers, and a number was allocated to each mother. After that, mothers were randomly included in the research according to their identification number through simple random sampling method.

Members of the statistical sample were then selected from this list using the table of random numbers. After identifying the research population, the research team contacted families using their phone numbers in the case files, invited them to the health center after introducing themselves and clarifying the research objectives, and distributed research questionnaires among the subjects after receiving their written consent forms.

### Study instrument

To achieve the research goal, the research data was collected by two methods. First, through the available information and documentation in the household files of families with ≤12-month-old children such as exclusive breastfeeding in the first 6 months of life, birth weight and similar cases; and second, through a researcher-made multi-item questionnaire examined in terms of design and psychometric aspects following desk studies, and searching the sources and Internet papers using similar studies [3, 10, 15, 17, 24–26]. A multi-part questionnaire consisted of the following sections: 1- mothers’ demographic data including their occupation, age, number of children, place of residence, and illness, and mother’s education, spouse’s education, and family income; 2- demographic data of children including gender, weight, height, length and weight, head circumference, disease, birth rank, and growth and development status; 3- pregnancy and childbirth factors including smoking, receiving prenatal education, unwanted pregnancy or planning for pregnancy, duration of pregnancy, type of delivery, multiple pregnancy, type of feeding immediately after delivery, infant admission to neonatal intensive care unit, type of care during pregnancy, regular care during pregnancy, and type of access to health volunteers; 4- mother’s knowledge on breastfeeding with 5 items; 5- mother’s attitude towards breastfeeding with 5 items; 6- perceived social support by three questions; 7- perceived self-efficacy with 10 questions; 8- intention and decision for breastfeeding with 2 questions, and 9- breastfeeding behavior with 6 questions.

The content validity of the questionnaire was confirmed using the qualitative and quantitative validity methods. We asked for the opinion of six specialists in the field of health education and promotion, epidemiology, public health, pediatrics, and midwifery. In the qualitative assessment, six specialists reviewed the questionnaire to check its grammar, use of correct words, right scaling and right placement of items. In quantitative method, both content validity ratio (CVR) and content validity index (CVI) were calculated. Regarding CVR, values > 0.56 were considered “acceptable”, and concerning CVI, values > 0.79 were considered acceptable [27].

Cronbach’s α was used to measure the internal reliability of the item. To this aim, the questionnaire was completed by 20 mothers. The reliability for the entire scale was (α = 0.792), covering attitude (α = 0.566), self-efficacy (α = 0.754), social support (α = 0.628), and breastfeeding behavior (α = 0.738).

### Statistical analysis

Structural equations model (SEM) was used to investigate the relationship between latent and observed variables. Latent variables are those not directly observed but extracted from other variables called indicators. In this study, we had five latent variables, including self-efficacy, social support, attitude, breastfeeding intention, and breastfeeding behavior. The indicator variables of these latent variables are presented in Table 1. SEM was established for a comprehensive assessment of the associations among latent variables. In the measurement part of the model, latent variables were linked to the corresponding indicator variables in a confirmatory framework based on the literature. In the structural part, breastfeeding behavior was considered as latent response, while self-efficacy, social support, and attitude were considered as latent predictors. Breastfeeding intention was considered in the structural part of the model as mediator. Potential confounders including previous breastfeeding experience, breastfeeding immediately after childbirth in hospital, the number of twins, number of children, mothers’ education and type of delivery variables were further considered in the structural part of the model as covariates. The fitting SEM was conducted by AMOS 18.0 software (SPSS, Inc., Chicago, IL, USA) and model parameters were estimated using maximum likelihood method. Model fit was assessed by the following indices associated with goodness of fit: The Tucker–Lewis coefficient (TLI), comparative fit index (CFI), root mean square error of approximation (RMSEA), χ2/df, normed fit index (NFI), and relative fit index (RFI). CFI, TLI, NFI and RFI values range from 0 to 1; values of 0.90 or above indicate acceptable fit. χ2/df should be as high as 5. The RMSEA value ranges from 0 to 1, with smaller values of this index indicating better model fit (28). Regression coefficients in the structural part of the fitted model were reported as the measures of associations. Quantitative and qualitative variables were expressed as mean (SD) and number (percentage), respectively.

**Table 1:** Variables Used in SEM Fitting

Dimension	Variable
social support	Breastfeeding recommendations given by close friends and relatives (q1) / conjugal support perception (q2) / perception of healthcare community workers’ support (q3)
self-efficacy	Sufficient milk diagnosis (q4), overcoming breastfeeding challenges (q5), exclusive feeding (q6), satisfaction (q7), determination on exclusive feeding (q8), satisfaction with breastfeeding experience (q9), combating time-consuming nature of breastfeeding by mother (q10), breast milk depletion (q11), breastfeeding on demand (q12), baby’s satisfaction with breastfed milk (q13)
attitude	Mother’s joy of breastfeeding (q14) / preferring powdered milk to breastfeeding for facility (q15) / considering breastfeeding as pleasurable (q16) / considering breastfeeding as more economical (q17) / considering mother’s milk as nutrient-richer (q18) / viewing breastfeeding to cause mother fitness (q19) / satisfaction with breastfeeding experience (q20)
intention	Planning for breastfeeding (q21) / perceiving fulfillment of exclusive breastfeeding terms and conditions (q22)
Breastfeeding behavior	Start of breastfeeding immediately after delivery (q23) / breastfeeding based on child’s demand or mother’s desire (q24) / using feeding bottle (q25) / using pacifier (q26) Baby’s past nutrition formula (q27) / nighttime feeding schedule (q28) / daytime feeding schedule (q29) / breastfeeding schedule basis (q30) / breastfeeding from both breasts (q31)

## Results

In the present research, all mothers accepted the research team invitation, but a total of 20 out of the 420 distributed questionnaires were rejected due to corrupted data and incompleteness; the final analysis was performed with 400 questionnaires. In the research, about two thirds (77%) of the studied mothers were housewives, and the household income of 51% was moderate and that of 30.5% was low. 86.8% had a 9-month pregnancy length, and 71% had one or two children. 5% of the children were twins. In terms of the type of delivery,50.3% had cesarean sections; 48.3% of the subjects were urban inhabitants, and more than half (55.8%) of the children were males (Table 2).

**Table 2:** Descriptive information of participants

Variable	N(%)
Mother’s job	
Housewife	308(77)
Working at home	19(4.8)
Working outdoors	53(13.3)
Studying	20(5)
Address	
City	193(48.3)
Village	207(51.7)
Mother’s disease	
Yes	53(13.3)
No	347(86.7)
Mother smoking	
Cigarette	1(0.3)
Hookah	2(0.5)
None of them	397(99.3)
Type of delivery	
Cesarean section	201(50.3)
Normal	199(49.7)
Number of simultaneous pregnancy	
Twin	20(5)
Singleton	380(95)
Hospital nutrition	
Breast milk	378(94.5)
Milk powder	21(5.3)
Other cases	1(0.3)
Admitted to NICU	
Yes	63(15.8)
No	387(84.2)
Under care	
Health Center	249(62.3)
Specialist office	146(36.5)
Midwife office	5(1.3)
Type of referral	
Regular	295(73.6)
Irregular	105(26.3)
Access to health volunteers	
No	218(54.5)
Seldom	63(15.8)
Partially	57(14.3)
Yes	61(15.3)
Use of milk glass	
Yes	214(53.5)
No	186(46.5)
Use of pacifier	
Yes	116(29)
No	284(71)
Child sex	
Boy	223(55.8)
Girl	177(44.2)
Number of children	
One	124(31)
Two	160(40)
Three	87(21.8)
Four or more	29(7.4)
Pregnancy length	
7 months	6(1.5)
8 months	47(11.8)
9 months	347(86.8)
Income	
Low	122(30.5)
Medium	204(51)
Good	53(13.3)
Very good	19(4.8)
Excellent	2(0.5)
Feeding with two breasts	
No	14(3.5)
Somewhat	46(11.5)
Yes	340(85)

**Fig. 1:**
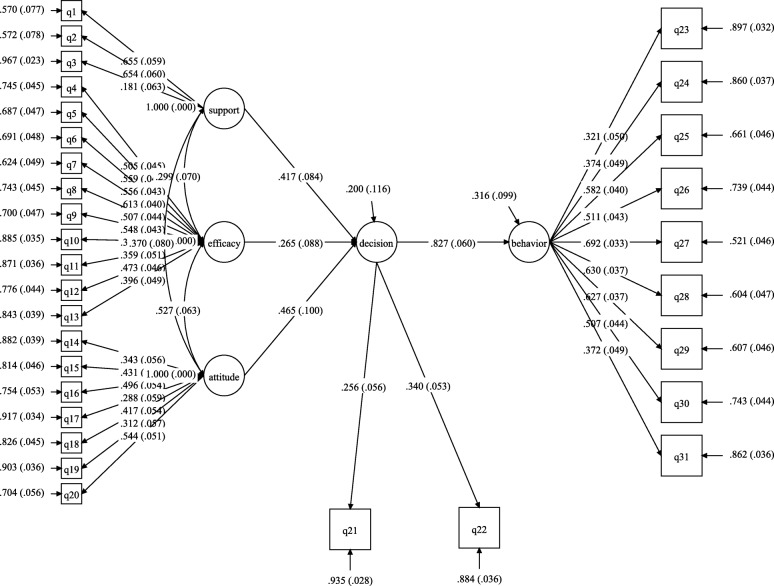
Association of self-efficacy, social support, attitude, and breastfeeding intention with breastfeeding behavior based on structural equation modling

To assess the association between breastfeeding behavior and self-efficacy, social support, attitude, and breastfeeding intention considering the impacts of potential confounders, a structural equation model was fitted, shown>/> in Fig. 1. In the measurement part of the model, latent variables (ovals) were linked to the related indicators (rectangles). In the structural part of the model, breastfeeding behavior as a latent response was regressed on latent predictor, meaning breastfeeding intention. In addition, breastfeeding intention, as mediator, was regressed on self-efficacy, social support, and attitude. Table 3 represents the unadjusted and adjusted models of the associations among the aforesaid variables. Both adjusted and unadjusted models had acceptable fitness based on model fitting criteria (unadjusted model: χ2/df = 2.205; NFI = 0.643; RFI = 0.586; RMSE = 0.055; CFI = 0.76; TLI = 0.74; adjusted model: χ2/df = 2.15; NFI = 0.604; RFI = 0.541; RMSE = 0.054; CFI = 0.713; TLI = 0.700). According to Table 3 and Fig. 1, statistically significant associations were observed between self-efficacy, social support, attitude, and breastfeeding intention and behavior. In unadjusted model, self-efficacy (β = 0.219; 95% CI: 0.079, 0.359), social support (β = 0.345; 95% CI: 0.214, 0.476) and attitude (β = 0.384; 95% CI: 0.224, 0.545) had indirect significant associations with breastfeeding behavior (*P* < 0.01). In addition, breastfeeding intention was positively and strongly associated with breastfeeding behavior (β = 0.827; 95% CI: 0.709, 0.945, *P* < 0.001). In the adjusted model, similar results were obtained through controlling the effects of previous breastfeeding experience, breastfeeding immediately after childbirth in hospital, the number of twins, number of children, mothers’ education and type of delivery. Mothers’ attitude (β = 0.442; 95% CI: 0.284, 0.599), self-efficacy (β = 0.186; 95% CI: 0.047, 0.324) and perceived social support (β = 0.178; 95% CI: 0.035, 0.322) were indirectly associated with breastfeeding intention (*P* < 0.05). Besides, the breastfeeding intention affected breastfeeding behavior with a high coefficient (0.857; 95% CI: 0.735, 0.979).

**Table 3:** Direct, indirect and research factors effects on the breastfeeding behavior

Model	Research Factors	Direct effect	Indirect effect	Total effect
Model1 (crude model)	Self-efficacy	–	0.219^b^ (0.079, 0.359)	0.219^b^ (0.079, 0.359)
	Perceived social support	–	0.345^a^ (0.214, 0.476)	0.345^a^ (0.214, 0.476)
	Attitude towards breastfeeding	–	0.384^a^ (0.224, 0.545)	0.384^a^ (0.224, 0.545)
	breastfeeding intention	0.827^a^ (0.709, 0.945)	–	0.827^a^ (0.709, 0.945)
Model2^a^ (adjusted model)	Self-efficacy	–	.186^b^ (0.047, 0.324)	.186^b^ (0.047, 0.324)
	Perceived social support	–	.178^c^ (0.035, 0.322)	.178^c^ (0.035, 0.322)
	Attitude towards breastfeeding	–	.442^a^ (0.284, 0.599)	.442^a^ (0.284, 0.599)
	breastfeeding intention	0.857^a^ (0.735, 0.979)	–	0.857^a^ (0.735, 0.979)

Values are regression coefficients (95% CI)

^a^ *P* < 0.001; ^b^ *P* < 0.01; ^c^ *P* < 0.05

^a^In model 2, the effects of previous breastfeeding experience, breastfeeding immediately after childbirth in hospital, the number of twin, number of children, mother education and type of delivery variables have been adjusted

## Discussion

The research results indicated that mothers’ attitudes toward breastfeeding had the maximum effect on their breastfeeding intention. From an analytical point of view, if mothers find breastfeeding a satisfying and pleasing practice, and believe that the breast milk is the best nutrition for infants up to 6 months, they will be more determined to provide exclusive feeding, which would ultimately affect their breastfeeding behavior. This finding is consistent with the results of Froehlich et al. [28], and Bich et al. [29] who found that attitude had a positive and significant effect on breastfeeding. Moreover, Kang et al. [30] observed the negative attitude toward breastfeeding as the most important cause of non-breastfeeding in Korean women, which is consistent with the results of the present research. However, health workers should note that attitudes are adventitious, and after experiencing a behavior, positive or negative beliefs about the consequences of behavior will be strengthened, later affecting the continuity of that behavior as an incentive; thus, positive attitudes towards breastfeeding should be strengthened.

The findings of the present study indicated that the intention was the most important predictor of breastfeeding behavior. Consistent with the present research, various studies have proven a strong relationship between intention and behavior; for instance, in a meta-analysis study by Guo et al. [31], the intention was a strong predictor for breastfeeding behavior. According to a meta-analysis by Armitage et al. [32], the behavior intention responded to 22% of behavior variance in 48 published papers on average. In a review study by Sheeran [33], the results indicated that the intention in the 82,107 studied samples responded to 28% of behavior variance on average.

A previous study indicated that the perceived social support was a social and facilitating psychological factor of health behavior able to solve behavioral problems and barriers in supported people and stabilize their proper behavior [34]. Our research further proved that the more the spouses and health volunteers encourage mothers to opt for exclusive breastfeeding, the more the mother’s intention for exclusive breastfeeding will increase, which is consistent with previous studies [27, 28, 35], indicating that social support is an important and predictive variable in health behaviors such as breastfeeding. Similarly, Giles et al. [36], mentioned the supportive roles of mothers, spouses, family, close friends, and health workers in the continuity of breastfeeding and stated that breastfeeding played more important roles in the start and end of the breastfeeding period, with training physicians and nurses also playing important roles in this regard.

Our results indicated that self-efficacy had a positive and significant relationship with breastfeeding intention and behavior, which is expected because previous studies such Wu et al. [37] and Brockway et al. [38] have proved the central role of self-efficacy as an important factor in the prediction of breastfeeding intention and duration. Breastfeeding self-efficacy is a psychological factor influencing the breastfeeding duration [15]. Researchers have proved that self-efficacy increases individual participation in proper health behavior and affects the levels of individual effort and performance [34].

Results of demographic variables indicated that there was a significant positive relationship between multiple-pregnancy and breastfeeding behavior and intention, which is inconsistent with the conclusion drawn by McDonald et al. [16] in Canada. Unlike the present research, they found that singleton children were more exclusively breastfed than twins probably due to the small number of twins in the present study compared with McDonald’s research. Future studies should investigate this relationship with larger sample sizes. Also, the number of children had a positive relationship with the mother’s intention for exclusive breastfeeding in the present study. Similar to the present study, Simard et al. and Adams [39] observed a significant relationship between mothers’ breastfeeding and the number of children, which could be attributed to previous experiences of mothers in breastfeeding or support for older children.

According to the present findings, natural delivery increases the chance of breastfeeding and its continuation, which is similar to the results of Zanardo et al. [40] who studied the relationship between delivery type and breastfeeding model in 2137 3-day-old infants. The amount of breastfeeding in the natural delivery group was significantly higher than the cesarean section, particularly following the 3-month and 6-month follow-ups. Evans [41] found that the chance of breastfeeding was reduced in the cesarean section for reasons such as later maternal healing as well as more pain and risks compared with normal delivery. In total, cesarean section prevents the onset and continuation of breastfeeding by reducing the mother’s sense of adequacy and ability (self-efficacy).

Another result of the present study was a positive and significant relationship between breastfeeding immediately after delivery and exclusive feeding, which consistent with the findings of a research in Taiwan, in which the delay in the onset of breastfeeding reduced the amount of exclusive breastfeeding [42]. Previous studies have indicated that early breastfeeding is the main factor affecting the onset of breastfeeding [43]. The WHO recommends mother-child skin contact and breastfeeding in the first 30 min of birth, effective in the success of breastfeeding [10, 15, 26]. The early start of breastfeeding creates a healthy and powerful relationship between mothers and children, hence the necessity of breastfeeding immediately after birth; a delayed onset of breastfeeding contributes to shortened breastfeeding duration. Assessment the informative pathways to breast feeding practice by using different health education and promotion theories are recommended for future researches.

Among the limitations in this research were the difficulty associated with obtaining breastfeeding information and lack of a complete response to the questions on the part of certain mothers. In addition, the study population of the current study was selected from one geographical region in Iran, reducing the generalizability of our results was reduced. The use of both household file information and questionnaire methods of data gathering was the strength of this study. In addition, the prediction model of exclusive breastfeeding behavior and intention provided a new classification of variables. Goodness of fit indices in the current study were not relevantly high, therefore the results should be interpreted with caution and it is suggested to conduct further studies with larger sample size.

## Conclusion

Our findings underscore the attention to mothers’ attitudes towards breastfeeding, their psychosocial and social support, and an ongoing encouragement by health workers, who should strengthen a sense of self-efficacy in mothers to start and continue breastfeeding. It is to be noted that most of the effective factors of breastfeeding, namely attitude, perceived social support, breastfeeding self-efficacy, the onset of breastfeeding and type of delivery at the hospital can be adjusted and modified by some interventions. We also recommend that family health system employees produce educational program about breast feeding practice based on the findings of the current study.

## Data Availability

The datasets used and analyzed during the current study are available from the corresponding author on reasonable request.

## Abbreviations

CFI: Comparative Fit Index
NFI: Normed fit index
RFI: Relative fit index
RMSEA: Root Mean Square Error of Approximation
SEM: Structural equation modeling
TLI: Tucker Lewis index
WHO: World health organization
